# Severe Hypogammaglobulinemia (IgG) During Efgartigimod Therapy in Neurological Practice: A Real-World Case Series

**DOI:** 10.7759/cureus.106599

**Published:** 2026-04-07

**Authors:** Patricio S Espinosa, Johanna Preisler, Ada Motiño Villanueva, McKenzie B Bellon, Charles H Hennekens

**Affiliations:** 1 Neurology, Espinosa Neuroscience Institute, Boca Raton, USA; 2 Neurology, Boca Raton Regional Hospital, Boca Raton, USA; 3 General Medicine, Universidad Católica de Honduras, Tegucigalpa, HND; 4 Neurology, Florida Atlantic University, Boca Raton, USA; 5 Preventive Medicine/Cardiology/Internal Medicine, Florida Atlantic University, Boca Raton, USA

**Keywords:** chronic inflammatory demyelinating polyneuroradiculopathy (cidp), efgartigimod, hypogammaglobulinemia, myasthenia gravis (mg)

## Abstract

Background

Efgartigimod is a neonatal Fc receptor (FcRn) antagonist approved for adults with generalized myasthenia gravis (gMG) who are anti-acetylcholine receptor antibody-positive, and for chronic inflammatory demyelinating polyradiculoneuropathy (CIDP). By blocking FcRn-mediated IgG recycling, efgartigimod lowers circulating IgG and reduces pathogenic autoantibodies. Clinical trials have demonstrated efficacy and an overall acceptable safety profile, but patients with low baseline IgG were generally not well represented.

Objective

The main objective of this study is to describe severe reductions in serum IgG observed in routine neurologic practice during efgartigimod therapy, and to explore their potential clinical implications, including infection risk and management considerations.

Methods

We performed a retrospective case series of 10 patients treated with efgartigimod in our practice between 2025 and 2026 for CIDP and MG. Available clinical data were reviewed, including diagnosis, prior immunotherapy exposure, baseline IgG (when available), serial IgG measurements, nadir IgG, and any documented infectious complications.

Results

Several patients developed marked hypogammaglobulinemia during treatment, including profound reductions to levels below 100 mg/dL in selected cases. Several patients reached IgG levels within ranges associated with increased susceptibility to infection (<500 mg/dL, particularly <400 mg/dL), although no severe infections were observed. One patient with CIDP and prior rituximab exposure had a baseline IgG of approximately 800 mg/dL, and later developed an IgG level of 66 mg/dL. Another patient without prior B-cell-depleting therapy also developed an IgG level below 100 mg/dL. No major infectious complication was documented during the observation period, but the degree of IgG suppression raised concern for clinically meaningful vulnerability to severe infection.

Conclusions

Efgartigimod therapy was associated with significant reductions in IgG in this real-world cohort. Because trial populations excluded or underrepresented patients with low baseline IgG, and did not meaningfully study extreme hypogammaglobulinemia, baseline and serial IgG monitoring should be strongly considered in all patients starting therapy, especially during the first six months. These findings highlight the potential for clinically meaningful IgG decline in certain patients, and support consideration of individualized monitoring strategies. These observations are hypothesis-generating and warrant further study.

## Introduction

Myasthenia gravis (MG) and chronic inflammatory demyelinating polyradiculoneuropathy (CIDP) are immune-mediated neurologic disorders in which IgG antibodies contribute importantly to disease pathophysiology. MG is a disorder of the neuromuscular junction, characterized by fluctuating, fatigable weakness, most often associated with autoantibodies against the acetylcholine receptor or other postsynaptic targets. CIDP is a chronic, immune-mediated neuropathy characterized by progressive or relapsing weakness, sensory dysfunction, and electrodiagnostic evidence of peripheral nerve demyelination. Although clinically distinct, both disorders frequently require long-term immunomodulatory therapy [1-3].

The primary objective of this study is to describe severe reductions in serum IgG observed in routine neurologic practice during efgartigimod therapy, and to explore their potential clinical implications, including infection risk and management considerations.

Efgartigimod is a human IgG1-derived Fc fragment engineered to antagonize the neonatal Fc receptor (FcRn). By blocking FcRn-mediated recycling of IgG, it accelerates IgG degradation and reduces circulating pathogenic autoantibodies. This mechanism has established efgartigimod as one of the major novel targeted therapies for antibody-mediated neurologic disease [1,2].

The clinical development program in generalized myasthenia gravis (gMG) demonstrated meaningful efficacy and supported regulatory approval. In the Phase 3 trial, Safety, Efficacy, and Tolerability of Efgartigimod in Patients with Generalized MG (ADAPT), efgartigimod improved disease-related outcomes in adults with gMG, particularly in anti-acetylcholine receptor antibody-positive patients [1]. Later, the Phase 3 study, Subcutaneous Efgartigimod PH20 in Generalized Myasthenia Gravis (ADAPT-SC), showed that the subcutaneous formulation was noninferior to intravenous efgartigimod with respect to total IgG reduction at Week 4, with a similar clinical profile. In that study, the mean total IgG reduction at Week 4 was 66.4% with subcutaneous treatment and 62.2% with intravenous treatment [2].

In CIDP, the Phase 2/3 ADHERE study further expanded the role of efgartigimod. In the randomized-withdrawal stage, relapse occurred in 27.9% of participants receiving subcutaneous efgartigimod, compared with 53.6% receiving placebo, corresponding to a hazard ratio of 0.39. Serious treatment-emergent adverse events in that stage occurred in 5% of both groups [3]. These results supported approval for adults with CIDP.

A central and expected feature of efgartigimod therapy is a reduction in total IgG. This pharmacodynamic effect is directly linked to its therapeutic mechanism. The prescribing information warns that Vyvgart may increase the risk of infection because of this reduction in IgG. In Study 1, the most common infections were respiratory tract infection and urinary tract infection [4]. At the same time, the published trial experience has generally been reassuring with respect to serious infection. However, these data must be interpreted carefully, because the drug remains relatively new in real-world practice, exposure outside trial settings is still limited compared with older therapies, and uncommon safety signals may not appear until larger numbers of patients are treated [4,5].

Although clinical trials have demonstrated an acceptable safety profile, their external validity may be limited in populations with baseline immunologic compromise, as patients with low baseline IgG or prior exposure to IgG-depleting therapies were typically excluded. In real-world practice, these patients may be more common and may experience more pronounced IgG reductions during FcRn inhibition [6,7].

This limitation is particularly important because real-world neurology patients are often more medically complex than those enrolled in pivotal trials. Many have prior exposure to rituximab, corticosteroids, IVIG, plasma exchange, or other immunosuppressive therapies, which may already reduce immune reserve before efgartigimod is started. In our practice, we observed patients treated with efgartigimod who developed severe hypogammaglobulinemia, including profoundly low IgG levels well below those meaningfully studied in the clinical trial program. Because very low IgG levels are generally associated with increased vulnerability to infection, these observations are clinically important, even in the absence of immediate infectious complications.

In this report, we present a case series of 10 patients treated in our practice between 2025 and 2026 for CIDP and MG, in whom serial IgG measurements demonstrated substantial declines, including severe hypogammaglobulinemia in selected cases. Our goal is to highlight this potential safety concern and emphasize the importance of baseline and serial IgG monitoring during treatment.

## Materials and methods

This study was conducted at the Espinosa Neuroscience Institute, Boca Raton, FL, USA, an outpatient neurology clinic. We performed a retrospective case series of patients treated in our neurology practice with efgartigimod between January 1, 2026, and March 30, 2026, for immune-mediated neurologic diseases, including CIDP and MG.

Patients were identified through a retrospective review of individuals treated with efgartigimod during the study period. Eligible patients consisted of consecutive individuals with a diagnosis of MG or CIDP, who presented for follow-up between January 1, 2026, and March 30, 2026, and received treatment with efgartigimod. Inclusion criteria included age ≥18 years, a confirmed diagnosis of MG or CIDP based on standard clinical criteria, receipt of at least one treatment cycle of efgartigimod, and availability of serum IgG measurements at baseline and during follow-up. Patients with insufficient laboratory data, absence of IgG measurements, lack of treatment with efgartigimod, or incomplete clinical records precluding outcome assessment were excluded.

Serum IgG levels were obtained as part of routine clinical care, typically including a baseline measurement prior to therapy initiation, and follow-up assessments during and/or after treatment cycles. The timing of follow-up measurements was not standardized, but generally occurred within several weeks after treatment initiation or at the time of clinical reassessment, reflecting real-world practice.

Clinical monitoring was performed according to standard care, and included routine evaluation for potential adverse effects and intercurrent infections. Patients were treated with efgartigimod according to routine clinical practice for their underlying neurologic condition.

Clinical data were collected through retrospective chart review and included demographic characteristics (age and sex), underlying neurologic diagnosis, prior immunotherapy exposure (including agents such as rituximab), baseline IgG levels, when available, nadir IgG levels, timing and magnitude of IgG changes, duration of treatment, and relevant clinical outcomes, including infectious complications and treatment-related adverse events.

A total of 10 patients met the inclusion criteria and were included in the analysis. Serial IgG measurements were obtained as part of standard clinical monitoring, rather than a predefined research protocol, and, therefore, the timing of laboratory assessments was not uniform across all cases. Despite this variability, the available data were sufficient to identify clinically meaningful IgG reductions, including cases of profound hypogammaglobulinemia.

The primary descriptive outcome was the magnitude of IgG decline during therapy. Secondary descriptive outcomes included the presence or absence of infectious complications and the potential contribution of prior immunosuppressive exposure, particularly rituximab or other therapies known to reduce humoral immune reserve.

Given the observational design, small sample size, and variability in laboratory monitoring, this study is intended to be descriptive and hypothesis-generating, rather than infer causal relationships.

## Results

A total of 10 patients treated with efgartigimod in our practice between 2025 and 2026 were identified as having available IgG data (Table 1). Diagnoses included CIDP and gMG. Several patients had prior exposure to immunomodulatory or immunosuppressive therapies, including rituximab.

**Table 1:** Clinical Characteristics and IgG Outcomes in Patients Treated With Efgartigimod Normal indicates baseline IgG within laboratory reference range: 600-1600 mg/dL. CIDP: chronic inflammatory demyelinating polyneuropathy; MG: myasthenia gravis; UTI: urinary tract infection

Case	Diagnosis	Age (yrs)	Sex	Baseline IgG (mg/dL)	Nadir IgG (mg/dL)	Duration of Therapy	Clinical Effectiveness	Infections	Hospitalization
1	CIDP	80	M	Normal	196	6-12 months	No	None	Yes (weakness)
2	CIDP	79	F	700	66	6 months	Yes	None	No
3	CIDP	74	M	870	277	>5 months	Yes	UTI ×3	No
4	CIDP	72	M	Normal	299	>6 months	Yes	None	No
5	CIDP	63	M	Normal	324	>6 months	No	UTI ×1	No
6	CIDP	57	F	Normal	299	>6 months	No	UTI ×1	No
7	MG	65	M	Normal	323	>12 months	No	COVID-19	Yes
8	CIDP	67	M	Normal	100	~5 months	Not stated	None	No
9	MG	82	F	Normal	213	6-12 months	Yes	None	No
10	CIDP	74	M	1200	323	>6 months	Yes	None	No

The most striking finding was the occurrence of profound hypogammaglobulinemia in selected patients. Several patients reached IgG levels below 300 mg/dL, including levels below 100 mg/dL, consistent with severe hypogammaglobulinemia.

In one patient with CIDP and prior rituximab exposure, baseline IgG was approximately 600 mg/dL before treatment, and subsequent testing revealed a nadir IgG of 66 mg/dL. This level was identified because IgG monitoring had been performed in the context of prior rituximab exposure, and borderline baseline IgG. In another patient without prior B-cell-depleting therapy, serum IgG also fell to 100 mg/dL during treatment, demonstrating that severe IgG suppression may occur even in the absence of prior immunosuppressive exposure.

Across the series, the extent of IgG decline varied by patient, but the overall pattern suggested that substantial suppression may occur in routine clinical practice, and may at times exceed the degree reported in clinical trials (Table 1). None of the patients experienced a documented major infectious complication directly attributable to therapy during the observation period. However, the depth of IgG decline in several cases raised concern for clinically meaningful vulnerability to infection, particularly with prolonged therapy, or in patients with additional risk factors.

Case descriptions

Case 1

An 80-year-old man with CIDP was treated with efgartigimod for approximately 6-12 months. His baseline IgG level was within normal limits, and the nadir IgG was 196 mg/dL. Therapy was discontinued due to a lack of clinical effectiveness and declining IgG levels. The patient did not develop infections, but required one hospitalization due to worsening weakness. He was subsequently transitioned to intravenous immunoglobulin (IVIG).

Case 2

A 79-year-old woman with CIDP was treated for six months. Her baseline IgG level was 700 mg/dL, and the nadir IgG was 66 mg/dL. Despite the profound IgG reduction, the patient did not develop infections or require hospitalization. Treatment was considered clinically effective.

Case 3

A 74-year-old man with CIDP was treated for more than five months. His baseline IgG level was 870 mg/dL, and the nadir IgG was 277 mg/dL. The treatment was clinically effective, with no worsening of neurologic symptoms; however, the patient developed three urinary tract infections during therapy.

Case 4

A 72-year-old man with CIDP was treated for more than six months. His baseline IgG level was within normal limits, and the nadir IgG was 299 mg/dL. The patient reported no infections or hospitalizations, and treatment was considered effective.

Case 5

A 63-year-old man with CIDP was treated for more than six months. His baseline IgG level was normal, and the nadir IgG was 324 mg/dL. The treatment was not clinically effective. The patient developed one urinary tract infection, but did not require hospitalization.

Case 6

A 57-year-old woman with CIDP was treated for more than six months. Her baseline IgG level was normal, and the nadir IgG was 299 mg/dL. The patient experienced a lack of treatment effectiveness and developed a urinary tract infection. No hospitalizations were reported.

Case 7

A 65-year-old man with gMG was treated for more than one year. His baseline IgG level was normal, and the nadir IgG was 323 mg/dL. The patient experienced recurrent exacerbations of weakness despite treatment. He was hospitalized due to COVID-19 infection and required intravenous steroids and IVIG.

Case 8

A 67-year-old man with CIDP was treated for approximately five months. His baseline IgG level was normal. His IgG declined to a nadir of 100 mg/dL, followed by recovery to 157 mg/dL and 265 mg/dL after discontinuation of therapy. The patient did not develop infections or require hospitalization.

Case 9

An 82-year-old woman with gMG was treated for approximately six months. Her baseline IgG level was normal, and the nadir IgG was 213 mg/dL, followed by a further increase to 275 mg/dL four weeks after discontinuation of therapy. The patient did not develop infections or require hospitalization. Treatment was considered clinically effective.

Case 10

A 74-year-old man with CIDP was treated for more than six months. His baseline IgG level was 1200 mg/dL, and the nadir IgG was 323 mg/dL. The patient did not develop infections or require hospitalization, and treatment was considered effective.

Statistical analysis

The mean nadir IgG level across patients was approximately 223 mg/dL. Overall, 4 out of 10 patients (40%) developed IgG levels below 200 mg/dL, and two patients (20%) had IgG levels below 100 mg/dL. Infections occurred in four patients (40%), primarily urinary tract infections, with one serious infection (COVID-19 requiring hospitalization). Overall, treatment effectiveness was observed in 6 out of 10 patients (60%).

## Discussion

Efgartigimod has rapidly emerged as an important therapy for antibody-mediated neurologic disease. The efficacy data in gMG and CIDP are strong, and the drug represents a major therapeutic advance for patients who require targeted immunotherapy [1-3]. At the same time, its mechanism of action intentionally lowers total IgG, and, therefore, concern about infection risk is biologically unavoidable [4].

FcRn plays a central role in regulating IgG homeostasis by protecting immunoglobulins from lysosomal degradation and extending their half-life. Therapeutic FcRn inhibition accelerates IgG catabolism, resulting in reduced circulating IgG levels in autoimmune neurologic diseases, and influencing the pharmacokinetics and clinical efficacy of IVIG, as FcRn blockade may shorten its duration of action. Our findings align with these mechanistic insights, as we observed that IgG suppression in routine clinical practice can be profound, with several patients developing severe hypogammaglobulinemia, including levels below 100 mg/dL. Notably, this occurred both in patients with and without prior exposure to B-cell-depleting therapies, suggesting that FcRn blockade alone may lead to clinically significant immunoglobulin depletion.

IgG reduction during efgartigimod therapy is an expected pharmacodynamic effect and is generally described as transient in clinical trials, particularly following interruption or completion of treatment cycles. However, in our series, some patients demonstrated marked declines, reaching severe hypogammaglobulinemia, with recovery that appeared prolonged or incomplete within the observed timeframe.

In clinical practice, IgG levels below approximately 500 mg/dL, and particularly below 400 mg/dL, are generally considered clinically significant and are associated with increased susceptibility to infections.

Although no major infectious complications were observed, the depth of IgG decline raises concern for increased vulnerability to infection, particularly with prolonged therapy or reduced immunologic reserve. Although no severe infections were observed, this may reflect the limited sample size, duration of follow-up, or proactive clinical management.

Additionally, the interaction with IVIG, described in prior literature, is clinically relevant, as accelerated IgG clearance may impact treatment strategies in patients requiring sequential or adjunctive immunotherapy. Variability in treatment response may also be influenced by genetic polymorphisms in the FCGRT (Fc fragment of IgG receptor and transporter) gene, which regulates FcRn expression and function. Taken together, these findings underscore the importance of individualized management in patients receiving FcRn-targeting therapies, such as efgartigimod.

In addition, efgartigimod represents an important and highly effective therapeutic option for patients with both MG and CIDP. Its targeted mechanism offers a meaningful advance in the treatment of antibody-mediated neurologic disease, providing patients with an alternative to traditional immunosuppressive therapies. By inhibiting FcRn, efgartigimod reduces the recycling of IgG and accelerates its degradation, thereby lowering circulating pathogenic antibodies. In MG, this includes the reduction of acetylcholine receptor antibodies that impair neuromuscular transmission. This mechanism directly contributes to clinical improvement by decreasing the autoimmune attack on the neuromuscular junction and peripheral nerves.

However, it is important to recognize that this therapeutic benefit is achieved through intentional immunomodulation. By reducing circulating IgG levels, efgartigimod effectively decreases the immune response, which is central to its efficacy, but also introduces a potential risk.

While IgG levels may recover after discontinuation of therapy, continued treatment may sustain or deepen IgG suppression, which could increase the risk of clinically relevant complications, including infections.

While clinical trials have demonstrated that reductions in IgG are expected and generally well tolerated, patients who develop very low IgG levels may be at increased risk of infection. This is particularly relevant in real-world populations, where patients often have additional risk factors, such as prior immunosuppressive therapy, advanced age, or comorbid conditions.

Clinical trial data have generally been reassuring. The ADAPT and ADHERE programs did not demonstrate a major signal for excess serious infection in the studied populations, and the prescribing information reflects a manageable, but real, infection warning rather than a prohibitive safety profile [1,3,4]. However, these findings should not be overgeneralized. Trial populations are selected, monitored closely, and often exclude patients with low baseline immune reserve.

Normal adult IgG levels vary somewhat by laboratory, but are commonly in the range of approximately 600 to 1600 mg/dL. Progressive concern arises as IgG falls below about 400 mg/dL, and profoundly low values, below 200 mg/dL, are associated with significantly impaired humoral immune protection. Values below 100 mg/dL are, therefore, highly concerning, even when the patient has not yet developed a documented serious infection.

Our cases are important because they suggest that, in routine clinical practice, some patients on efgartigimod may develop IgG levels far below those meaningfully characterized in published trials. One patient in our practice reached an IgG level of 66 mg/dL, and another patient without prior rituximab also fell to 100 mg/dL. Although none of our patients developed a major infection during the observation period, the absence of immediate clinical harm does not eliminate concern.

The clinical relevance of these findings lies in identifying patients who reach IgG levels known to confer increased infection risk, supporting a proactive approach focused on monitoring and risk mitigation, rather than reactive management after complications occur.

Another important point is that our observations likely would have been missed without intentional monitoring. In one patient, severe hypogammaglobulinemia was recognized because baseline concern already existed due to prior rituximab exposure and an IgG level of around 600 mg/dL. Without active surveillance, profound reductions may go unnoticed until a patient presents with infection or another complication.

Baseline IgG assessment and periodic monitoring may be considered, particularly in patients with lower baseline levels or prior immunosuppressive exposure, to help identify those at risk for more pronounced declines.

Limitations

This study has several important limitations that should be considered when interpreting the findings. First, the small sample size limits generalizability and precludes robust statistical analysis. Second, the absence of a control group prevents direct comparison. Third, the observational case-series design does not allow for causal inference. Additionally, variability in the timing of laboratory assessments reflects real-world practice, rather than a standardized protocol. Accordingly, these findings should be considered descriptive and hypothesis-generating.

## Conclusions

Efgartigimod is an effective and important therapy for gMG and CIDP, but its therapeutic mechanism requires the reduction of circulating IgG. In this real-world case series, efgartigimod therapy was associated with significant reductions in serum IgG, including levels consistent with severe hypogammaglobulinemia. In our cohort, several patients developed marked IgG suppression, including profound reductions below 100 mg/dL and one case reaching 66 mg/dL. While causality cannot be established, these findings highlight the potential for clinically meaningful IgG decline in certain patients. Although no major infections were documented during the observation period, such levels are biologically concerning and may indicate increased vulnerability to infection, particularly in medically complex patients or those with prior immunosuppressive exposure.

These observations support consideration of individualized monitoring strategies, particularly in higher-risk populations, and suggest that clinically significant IgG reduction during efgartigimod therapy may be underrecognized in routine practice. Further investigation in larger, prospective studies is warranted to better define the incidence, clinical implications, and optimal management of IgG suppression, including whether specific thresholds should inform treatment modification, and how best to balance therapeutic benefit with infection risk.
